# New health taxes in Ghana: a qualitative study exploring potential public support

**DOI:** 10.1093/heapol/czaf042

**Published:** 2025-06-30

**Authors:** Katherine E Smith, Mark Hellowell, Divine D Logo, Robert Marten, Arti Singh

**Affiliations:** Centre for Health Policy, Department of Social Work & Social Policy, University of Strathclyde, Lord Hope Building, 141 St James Road, Glasgow G4 0LT, United Kingdom; Global Health Policy Unit, Social Policy, School of Social & Political Science, University of Edinburgh, Chrystal Macmillan Building, 15a George Square, Edinburgh EH8 9LD, United Kingdom; Ghana Health Service Research and Development Division, P.O. Box MB-190 Accra, Ghana; Alliance for Health Policy and Systems Research, World Health Organization, Geneva 1211, Switzerland; School of Public Health, KNUST, Kumasi, Ghana

**Keywords:** taxation, excise, Ghana, unhealthy commodities, alcohol, tobacco, sugar-sweetened beverages

## Abstract

In the context of a fiscal crisis and health pressures, Ghana’s government has been exploring additional pro-health taxes. The World Health Organization and World Bank support health taxes as ‘win-win’ policies that can, if designed effectively, simultaneously improve health and raise revenue for health spending. However, international evidence shows that health taxes can meet political and public opposition. Yet, there is little research that empirically examines public views of health taxes. We compared policy stakeholders’ *perceptions* of Ghanaian public support for health taxes with public views, seeking to understand the basis for potential public opposition, the extent to which evidence can shape public views, and whether tax framing and design influences public support. We undertook 28 semi-structured key informant interviews with stakeholders (from government, advocacy, and business groups) and five focus groups with 38 members of the public (purposefully selected for diversity in gender, age, ethnicity, occupation, and social background). We employed an innovative deliberative design for the focus groups, which enabled us to explore how public views responded to contrasting health tax ‘frames’. Stakeholders generally believed public support for health taxes was low, especially for more widely consumed products. Yet, most focus group participants expressed strong support for health taxes, *especially* those targeting (more widely-consumed) sugar-sweetened beverages. Support increased when health taxes were framed as measures to improve public health and/or create a fairer tax system, and when commitments were made to using resulting revenue for health spending (known as ‘earmarking’ or hypothecation). However, stakeholders and members of the public shared a concern that business influence in Ghanaian politics presents a key barrier to implementing effective health taxes sustainably. Overall, our findings suggest that health taxes with a clearly-framed health rationale could command strong Ghanaian public support but likely require effective advocacy to overcome political barriers.

Key Messages

- Ghana is considering using further taxes to improve health (by reducing consumption of unhealthy products and obtaining revenue to support healthcare spending); a move supported by key global organizations and local civil society groups.
- Reflecting industry framing, most policy stakeholders interviewed believed public support for health taxes in Ghana was low and this perception appeared to be a barrier for action to introduce or advocate strongly for health taxes.
- Focus groups, however, suggest that when provided with basic information, the public is supportive of health taxes, and this support increases when taxes are framed as ‘win-win’ policies that can help improve heath, protect young people, ‘save lives’ and fund health services.
- These findings imply that there may be greater opportunities to introduce (or extend) health taxes in Ghana than many stakeholders believe, especially if proposals for health taxes are designed and framed as health measures.

## Introduction

Ghana faces a pressing need to tackle a growing burden of non-communicable diseases (NCDs). The World Health Organization (2022) estimated that NCDs accounted for nearly 45% of Ghana's all-cause mortality. Premature death (between 30 and 70 years) due to NCDs is 21% in Ghana (higher than the global average of 18%; WHO 2018). Unhealthy commodities, such as alcohol, sugar, and tobacco, are an important factor in this growing NCD burden. Moreover, health system strengthening efforts, likely to improve the ability of Ghana’s health system to respond effectively to NCDs, have been curtailed by the economic impacts of the COVID-19 pandemic [Ghana is one of the few countries in which per capita health spending was lower in 2022 than in 2019 (Kurowski *et al*. 2023)]. In this context, there has been renewed interest in exploring health taxes (Singh *et al*. 2023), with several calls by Ghanaian civil society organisations (CSOs) to implement taxes on unhealthy commodity industries, such as alcohol, sugar-sweetened beverages (SSBs), and ultra-processed foods (VALD 2020, A4H 2024).

*Ad valorem* (proportional) excise taxes are already levied on cigarettes and alcoholic beverages and, in 2021, the government of Ghana introduced the COVID-19 Health Recovery Levy Act, 2021 (Act 1068), which includes a 1% levy on the (in country) supply and import of goods and services (other than those detailed as exempt, such as live animals, goods for the disabled, educational items, and medical supplies). Ghana also recently passed: the Excise Duty Amendment bill 2022; the Growth and Sustainability Levy bill; and the Income Tax Amendment bill 2022. The Excise Duty (Amendment) Act, 2023 (Act 1108) (implemented from January 2024 onwards) includes an increase in excise duty on tobacco products, wine, malt drinks and spirits, and introduces an excise duty on SSBs (including fruit juices), electronic cigarettes and electronic liquids.

Health taxes like these have the potential to mitigate financial and health pressures because taxes on unhealthy commodities, such as tobacco, alcohol, and SSBs, are simultaneously one of the most effective ways of reducing consumption and a means of increasing revenue for health spending (Wright *et al*. 2017). However, attitudes toward such taxes vary (Wright *et al*. 2017, Lauer *et al*. 2023). International organizations, such as the World Health Organization and World Bank, tend to focus on health taxes’ potential to reduce unhealthy commodity consumption or stimulate reformulation (e.g. reducing the sugar content of drinks) (Lauer *et al*. 2023). However, national policy interest is often driven more by opportunities for generating additional revenue (James *et al*. 2020, Singh *et al*. 2023). Indeed, concerns are already being raised that Ghana’s expanding use of health taxes might prioritize increasing the country’s tax-to-GDP ratio over strategic opportunities to improve health (Asiedu 2023).

Public opinion (and policymakers’ perceptions of public opinion) can play an important role in the viability of health tax proposals (World Bank 2020, Lauer *et al*. 2023). Indeed, the Kenyan government recently decided to drop a series of planned tax increases in the context of extensive public protests (Rukanga 2024). While many of the proposed taxes in Kenya targeted essential products and were designed to raise revenue (rather than achieve health goals), it is a timely reminder of the importance of ensuring public support for new taxes.

The evidence base exploring public views of health taxes suggests that there are substantial variations by context and specific proposal. While some studies find relatively low public support for health taxes (González-Zapata *et al*. 2010, Barry *et al*. 2013, Beeken and Wardle 2013), recent survey data across five countries (Colombia, India, Jordan, Tanzania, and the USA) indicated that people are largely supportive of instituting taxes for better health outcomes (Dugan 2022).

A recent nationwide poll that sampled 7794 Ghanaian residents found that a 84.3% of respondents were concerned about obesity and NCDs and that 67.7% would support government efforts to impose taxes on health-harming products (A4H and HD4HL Coalition 2022, Laar *et al*. 2023). When the survey proposed that the revenue generated by such taxes would be used to support public health interventions, support increased to 77.4% (A4H and HD4HL Coalition 2022, Laar *et al*. 2023). We were not able to identify any research using methods other than surveys to explore public views of health taxes in Ghana.

Our study builds on this opinion poll survey in two key ways: First, we comparatively assess policy stakeholder beliefs about public opinion on health taxes in Ghana with views of members of the Ghanaian public. This comparison is important because policy decisions are often informed more by policymakers’ perceptions of public opinion than by empirical evidence of public views, which can lead to unpopular policy decisions (see, e.g. Broockman and Skovron 2018). Second, we used deliberative focus group discussions to explore public views in a more in-depth manner. Since evidence shows that public opinions about health taxes are open to change (Kane and Malik 2019, James *et al*. 2020), and since governments are well placed to communicate the case for new policies to members of the public, the focus groups included a short, basic overview of NCDs and the potential impacts of health taxes.

## Methods

Our focus group design was informed by two distinct bodies of scholarship. First, deliberative methodologies, which propose that, when assessing public opinion about complex policy matters, it makes more sense to expose participants to evidence and allow people to form *reasoned* judgments than to simply ask uninformed citizens for spur-of-the-moment opinions (Degeling *et al*. 2015). Second, scholarship on policy ‘frames’ highlights that the way policy issues and proposals are articulated plays a role in shaping how they are understood (Weiss 1989). Since evidence specifically suggests that understanding frames and their construction is helpful in analysing how health taxes are advanced and thwarted (Koon and Marten 2023), we wanted to explore how members of the public responded to, and employed, common health tax ‘frames’.

Before engaging with members of the public directly, we wanted to understand how policy stakeholders *perceived* public opinion on health taxes so we conducted key informant interviews with policy stakeholders, supplementing these data with analysis of policy documents, grey and academic literature. We defined ‘policy stakeholders’ as individuals with an interest in health taxes stemming from their professional position. Specifically, we recruited politicians and policymakers involved in health and economic policy, relevant businesses, and CSOs in health (Table 1). We tailored our questions to interviewees’ expertise (Supplementary File). Next, to explore public views empirically, we used deliberative focus group discussions. Our deliberative design meant that, we were not seeking to elicit people’s immediate (unconsidered) reactions to questions about health taxes (which have already been explored via a recent opinion poll—Laar *et al*. 2023). Rather, we were seeking to explore how people’s views evolved in response to public health information (Supplementary file), contrasting health tax ‘frames’, and during discussion with others.

**Table 1.** Stakeholder organisations in the study.

Type of organization
Government (*n* = 9)
Civil society organizations (CSOs) (*n* = 4)
Media (*n* = 2)
International organizations (4)
Industry (*n* = 9)

The Committee on Human Research Publication and Ethics of KNUST, Ghana, granted ethics approval in January 2022 (CHRPE/AP/445/21).

### Stakeholder views

We purposively recruited 31 stakeholders from five sectors, of whom 28 agreed to participate (Table 1). Stakeholders were identified from an existing list of individuals involved in national level meetings and workshops on health taxes in Ghana. We stopped collecting new data when we felt we had reached a ‘saturation point’; a common method employed in qualitative research to guide sample size (Hennink *et al*. 2017). In our case, after 26 interviews, we felt we were encountering similar views within each sector (‘sample saturation’) and that the interviews contained sufficient information to enable us to interpret the reasoning underlying these views, and the variation in views across sectors (‘meaning saturation’) (Hennink *et al*. 2017).

All stakeholders were contacted via the WHO country office, using an official email, which was subsequently followed up with a phone call to confirm participation. Semi-structured interviews were conducted with each participant in English and/or Twi between June-October 2022, with each interview lasting 40–60 minutes. Interviews were conducted by Authors 3 and 5, face-to-face (*n* = 15 participants) or online (*n* = 4 participants) and online group interviews (*n* = 2). Although most interviews were conducted on a one-to-one basis (as was our intention), industry representatives insisted on group interviews so, for this sector, two group interviews were conducted, involving a total of nine participants. Online connectivity issues impacted three of the four online interviews (one with a CSO representative, one industry group and one with an interviewee from the Ministry of Health). We resolved these issues by contacting the interviewees on the phone to clarify any missing or unclear statements.

An interview guide was informed by the objectives of the larger study (Supplementary file), analysis of relevant published literature and policy documents. The stakeholder interviews are analysed in detail elsewhere (Singh *et al* 2023); in this study, we are focusing exclusively on a sub-section of these interview data relating to stakeholder perspectives of public views.

All interviewees received written information explaining the objectives of the study in advance and consented to participate in writing; all interviewees except industry actors agreed to be audio-recorded (where we instead took extensive notes). To allow interviewees to share their views as freely as possible, we offered interviewees anonymity and did not record any personal identification.

### Public participants and recruitment

We conducted five focus groups: two with men aged 16–34 years; one with women aged 16–34 years; one with men aged over 34 years; and one with women aged over 34 years. Although small scale, we used purposive sampling to ensure we were capturing a diversity of views in terms of gender, age, ethnicity, occupation, and social backgrounds (Table 2).

**Table 2.** Demographic details reported by participant.

Characteristics	*N* (%)
Gender	
Male	23 (65.8%)
Female	15 (39.5%)
Religion	
Christian	37 (97.4%)
Muslim	1 (2.6%)
Age range	
16–24	22 (57.9%)
25–34	4 (10.5%)
35–44	5 (13.2%)
45+	7 (18.4%)
Ethnic background	
Akan	12 (31.6%)
Ga	15 (39.5%)
Ewe	11 (28.9%)
Occupation	
Student	11 (28.9%)
Self-employed	11 (28.9%)
Trader	4 (10.5%)
Civil servant	3 (7.9%)
Beautician/seamstress	2 (5.3%)
Unemployed	7 (18.4%)

Participants were selected from the Achimota town Accra Metropolitan District. This is home to Alogboshie, a low-income, diverse community. We started by visiting a local Assemblyman (an elected official representing the Alogboshie community) to seek permission to conduct the study. After explaining the study objectives, young adults were recruited from the community by personally contacting them with the help of the Assemblyman. A snowballing approach was also used to recruit participants from diverse occupational and educational backgrounds and across age groups. Close to half of the focus group participants were identified via snowballing (Table 2). All focus groups were held in a school classroom in the community in August 2022 and lasted between 60 and 90 minutes. Those who agreed to participate were given an inconvenience allowance of GHC50 (USD10) to cover travel and other minor expenses.

The focus group discussions were facilitated by Author 3, who had prior experience in moderating focus groups and who worked to ensure that the approach was inclusive, creating opportunities for quieter participants to express their thoughts. The topic guide for the focus groups was initially developed by Author 1, informed by existing literature on public attitudes and experiences on health taxes (Farley *et al*. 2015, Wright et al. 2017, Eykelenboom et al. 2019). It was reviewed by Author 5 and refined by testing with a pilot focus group (see Supplementary File for final focus group topic guide).

Each focus group began with a brief introduction of participants, followed by a 5–10 minute PowerPoint presentation (Supplementary file) comprising basic health-related information on SSBs, alcohol, and tobacco consumption, and outlined the health rationale for raising taxes. This reflected our deliberative design and was intended to enable reasoned discussions as well as to avoid a situation in which those who felt they already knew a lot about health taxes dominated discussions. It also reflects an assumption that any new health taxes, or tax increases, would be accompanied by some level of public information regarding the rationale for the taxes. The main topics covered in discussions were: introduction of, and initial views about, health taxes on unhealthy products; views on the consumption of tobacco, alcohol and SSBs; the perceived role of health taxes in achieving behaviours change; debates around whether health taxes are fair; and perceptions of the role of government and advocates in introducing health taxes. In providing this information, we were seeking to mirror the kind of information that the Ghanaian Government and health stakeholders might reference in support of new (or increased) health taxes; this effectively provided a public ‘health’ frame for such taxes. Our deliberative design necessitated that we balanced this by explaining counter perspectives, so we also drew on our stakeholder interviews to explain that affected industries argue that health taxes are already adequate and that increasing or expanding health taxes would be likely to lead to more smuggling and illicit trade. This ensured that participants were also exposed to the more negative ways framing health taxes that affected industries tend to promote (Koon and Marten 2023). The group discussions were designed to facilitate deliberation about these alternative ways of thinking about, and framing, health taxes, which was further supported through the facilitation, which actively encouraged participants to consider alternative perspectives.

### Data analysis

The focus group discussions were digitally audio-recorded and audio files were uploaded to a secure server immediately after data collection. Authors 3 and 5 transcribed each interview and focus group discussion *verbatim* using Microsoft Word and removed identifying information. Authors 1, 3, and 5 reviewed transcripts for completeness and quality assurance, before deleting all audio files, in accordance with research ethics approval.

To analyse the coded interview data, we applied a framework approach (Smith and Firth 2011). This is a form of thematic analysis which encourages a step-by-step analytic approach, accompanied by an effective and transparent audit trail (Ritchie and Spencer 2002). The framework approach includes data familiarization, identifying a thematic framework, indexing, charting, and mapping/interpretation. We employed a primarily deductive approach to this analysis, with Authors 1 and 5 developing an initial coding scheme, informed by our study objectives (Supplementary file), relevant literature and documents. This included the two contrasting ways of framing these taxes that we had exposed participants to (i) as ‘win-win’ health policies that reduce the negative impacts of health harming products while raising revenue for health spending and (ii) a risky policy option that may increase smuggling and encourage illicit products. However, where we identified additional themes of relevance to the overarching project, we developed new codes in response (e.g. codes capturing non-tax interventions that participants suggested should be considered to reduce the health harms of alcohol, tobacco and SSBs). We also felt our initial binary way of framing health taxes did not adequately capture the variety of frames that participants introduced so we also developed new codes for the more specific frames that we identified within our data. All stakeholder transcripts were coded by Author 5, using NVivo qualitative analysis software (V.12) and a sample of 25% were cross-checked by Author 1. After this, Author 5 coded the focus group transcripts in NVivo. The final data interpretation was discussed among all authors. Our interpretation of stakeholder interviews was sense-checked with three key informants from the CSO and Government sectors.

## Results

We first outline stakeholder perspectives of public views about health taxes, before exploring the perspectives articulated in the focus group discussions with members of the public.

### Stakeholder perceptions of public views about health taxes

Across the data, stakeholder perceptions of public views on health taxes were mixed but the dominant view (in line with some existing international literature—Wright *et al*. 2017, Hoskins *et al*. 2019) was that public support for new, or additional, health taxes was likely to be low. For example, several stakeholders argued that members of the public would be hesitant about taxing unhealthy products because of their association with traditional practices (e.g. offering alcoholic beverages at marriage, naming ceremonies, and funerals). This is illustrated in the following quote, in which the speaker suggests some cultural changes will be required to engender public support:

[T]he dowries that we pay when we are going to marry, tobacco is something that you have to bring, strong drinks are something that you have to bring, they become part of the culture “…” So we have to look at this culture… (Ministry of Finance)

Reflecting this, there was a tendency for stakeholders to believe that people are more likely to support taxes on products they do not commonly consume. Since alcohol consumption is relatively high in Ghana, compared to tobacco (Boachie *et al*. 2022a, 2022b), several stakeholders suggested that public support would be lower for alcohol taxes. For example:

We consume a lot of alcohol in this country “…” People will support the taxes that they are not guilty of and oppose the one that they are guilty of, so somebody may support the taxes on tobacco because they don’t smoke but frown on same on alcohol because they drink (Ministry of Health)

In response, most interviewees suggested there was a need for consumer education to build public acceptance before any taxes could be introduced/increased. Some respondents in CSOs, such as the following interviewee, believed that consumers would, with the right information, support health taxes:

Talking about the public, you just need “…” education and information. Once they are well informed and sensitized on the dangers “…” and also the benefits of taxing the product and what the fund will be used for, I think the public will go for it (CSO with alcohol focus)

In contrast, industry stakeholders suggested that health taxes should be avoided, and that efforts to raise consumer awareness should focus on achieving reductions in consumption via behavioural changes (independently of taxation). This viewpoint reflects wider research identifying a tendency among producers and retailers of unhealthy products to advocate against taxation (Wright *et al*. 2017) and to promote (less effective) education-led behaviour change instead (Landman *et al*. 2002, Balbach *et al*. 2006, Young *et al*. 2018).

We need more education and awareness of public, let people make their own informed choices. (industry)

In summary, although some stakeholders (mainly in health focused CSO organisations) believed that public support for health taxes could be achieved, the consensus was that work would be required to achieve such support (e.g. educational campaigns and changes to cultural practices). There was also a widely held view that people would be less likely to support taxes on products they regularly consume or on products associated with traditional cultural practices (alcohol taxes, in particular, were deemed likely to meet public opposition due to their role in marriages, funerals, and other activities).

### Public perspectives on health taxes

The data we gathered from focus group discussions contrasts with the stakeholder perceptions, showing strong (almost universal) support for health taxes among our participants. While there was some variation across products, support was the highest for the products which focus group participants said they most commonly consumed (SSBs and other sugary products), followed by alcohol, then tobacco. This was the opposite of stakeholder expectations.

In the following sub-sections, we explore: the reasons participants provided for supporting health taxes; their concerns about, and potential objections to, health taxes; their views about the potential role of advocacy and public campaigns in shaping public support; the ways in which framing health taxes appeared to influence public support; and views on heath taxes design.

### Reasons for supporting health taxes

By far, the most popular reason given for supporting health taxes was the perceived effectiveness of such taxes in reducing consumption and the benefits of this for individuals in terms of their health and finances. The following are just a small illustration of the consistency of this perspective, which was articulated in all focus group discussions:

Focus Group 1 (men 16-34) participant: *[A]lcohol drinks affect us “…” so they should put the taxes on it to increase the prices it will help society. “…” If the prices of a bottle is increased from… GHC5 [USD0.33] to GHC20 [USD1.31], it will stop us from buying and save us from many problems.*

Focus group 2 (women 16-34) participant: *I have just realized [in response to presentation] that [sugar sweetened drinks] will harm me in the future “…” so the taxes will help me stop buying and save money. “…” If I don’t get the money to go and buy, it will help me in so many ways.*

Focus group 4 (women 35+) participant: *It is fair because if the taxes are placed on the products and you can’t buy, you will stay away and save your life.*

Although participants were not asked to assess health taxes against other policy options that had been advocated by some stakeholders (e.g. educational campaigns), participants’ discussions on addiction suggested they viewed taxes as more effective than education alone. For example:

Focus Group 1 (men 16-34) participants: I like these sugary drinks “…” so if the taxes are high and it have significant effects on the prices, it will help me “…” stop the bad habit of drinking.

Focus Group 5 (men 16-34): *I’m an addict to the sweet drinks so putting taxes on it, will “…” push me to stop consuming.*

The next most common reason for supporting health taxes related to the potential to raise revenue, especially for health spending (which had been briefly mentioned in the presentation—see Supplementary File), as the following extracts illustrate:

Focus group 2 (women 16-34) participant: *It will help us save a lot and get a lot the government to put mare infrastructure up and also get money to support the health insurance.*

Focus group 3 (men 35+) participant: *There are people in the villages who don’t have “…” access to medicine “…” so if the taxes are “…” used to help with building more hospitals and other things that may enhance the health of the people, and to support the health insurance, I may think it is fair.*

In three focus groups, participants supported health taxes as a ‘fair’ response to the health harms caused by these products, noting that those consuming more of these products were likely require greater support from publicly funded services, especially health services. For example:

Focus group 2 (women 16-34) participant: *The producers know their products are harmful and causing harm, so if the government goes ahead to tax the products, and effectively use the money, I think it is fair.*

Focus group 3 (men 35+) participant: *If there is no health tax, the population that has these products, if they get sick, they put so much pressure on the health system and families, and government… is not getting enough revenue from these products “…” I support the health taxes, so that the government can get some revenue to help.*

In three focus groups (1, 4, and 5), participants suggested that health taxes could help prompt positive societal shifts towards healthier norms. For example:

Focus Group 1 (men 16-34)*: It is right to put the taxes on these products to reduce consumption because “…” the soft drinks are drinks we see as normal to take “…” Societies across the country accepts it, today “…” but we have to know that so much of the intake has side effects.*

Focus Group 4 (women 35+): *if the taxes are placed on these products, it will help change the behaviour of many people “…” so slowly people may stop buying and consuming these products.*

While in focus groups 3 and 5, some participants suggested that health taxes might encourage positive shifts in corporate behaviour (e.g. by discouraging investments in the unhealthy commodity industries or by encouraging product reformulation):

Focus Group 3 (men 35+) participant: *We need to get back again to the solution, which is taxation. And then increase the taxation to such a rate that will deter the industry from venturing into such businesses.*

Focus Group 5 (men 16-34): *It will be wise to have the health taxes, to raise prices for the people to reduce or stop buying. If they realise that the sales are going down, the companies may “…” choose to change what they are producing, and produce healthy products.*

### Concerns about, and potential objections to, health taxes

Despite general support for health taxes, participants expressed concerns about implementation, particularly regarding how tax funds would be collected and spent. These concerns included potential implementation failures, a lack of transparency surrounding government spending, and the belief that wealthy individuals evade taxes.

Focus Group 5 (men 16-34): *We have policies, but implementing these policies, that is where the problem is “…” If people have money, they use their money to influence everything, especially the FDA [Food and Drugs Authority] - when the rich man goes there, they use their money to influence them to get their way.*

Focus Group 4 (women 35+) participant: *[I]f [the government] will do something good with [tax revenue], they would have done it, though I will support the taxes [but] I don’t think they will use it for anything good.*

As the above extracts illustrate, concerns around the effectiveness of implementation were often justified by arguing that the government had not achieved enough with existing taxes to necessarily justify more. This was commonly linked to a belief that bribery and corruption have the potential to undermine the effectiveness and fairness of any taxation plans. This prompted a few suggestions for measures that might accompany any new (or additional) health taxes, such as, ‘*committees that will make sure that these policies are “…” adhered to, so that they will do the right thing and people may not… bribe them’* (focus group 2 participant, men 16-34).

Next, while most participants seemed to believe that health taxes would reduce consumption, some questioned this. This apprehension partially related to some of the concerns about implementation and the potential for tax avoidance (as discussed above) but also stemmed from some concern that people with addictions may struggle to reduce their consumption and that people may find ways around health taxes, such as product switching or buying illicit products:

Focus Group 3 (men 35+) participant*: if they do it in that way, it is going to create a situation whereby the addiction is going to shift. “…” If I am addicted to alcohol and the taxes are higher on the alcohol, and low on sugars, I may shift to the sugars. “…” So… you are just shifting the problems but you are not solving it.*

Focus Group 4 (women 35+) participant: *The taxes can be raised, but we Ghanaians are something else. “…” Some may accuse the government of raising the taxes too high and then use the back door to produce fake ones into the system.*

In both the above examples, these concerns did not appear to undermine support for health taxes and, rather, participants proposed complementary measures to address these concerns. In the first example, this participant proposed that taxes should therefore be applied across unhealthy commodity products (to avoid the potential for people to switch to cheaper options) and, in the second, the participant went on to propose increased Food and Drugs Authority checks to combat the growth of the illicit market.

A minority concern, mentioned in just two focus groups (3 and 4), was a more principled objection to the idea of health taxes creating a situation in which some people suddenly cannot afford products they are used to consuming. For some participants, this seemed unfair. For example:

Focus Group 3 (women 35+) participant: *If they increase it too high and one cannot afford but others can, then that… will be discriminatory*

Finally, the following two concerns arose in just one focus group each: (i) the potential for negative economic impacts to arise from new or higher health taxes; (ii) a belief that, if unhealthy products are leading to the kinds of negative consequences outlined in the presentation, they should be just banned:

Focus Group 5 (men 16-34): *Putting too much cost on it will lead to unemployment, because “…” the companies are going to lay off workers. “…” So the taxes shouldn’t be too much.*

Focus Group 2 (women 16-34) participant: *I think they should stop doing all these bad products because even if they put the taxes on them, it will not be enough “…” so I think they should stop the production of these products.*

The idea that some products, especially tobacco, should be banned came up in several other focus groups (and is discussed further below) but it was only in Focus Group 2 that this was linked to a critique of health taxes.

### Views about the potential role of advocacy and public campaigns

Despite participants in all focus groups expressing support for health taxes, there was a strong consensus, across four of the five focus groups (all except focus group 2) that any efforts to introduce new or increased health taxes would require advocacy and public education campaigns to garner sufficient support. On this issue, the public focus group and stakeholder views aligned:

Focus Group 1 (men 16-34) participant*: A lot of us don't know the side effect of all these products, so we need to be educated. “…” I think when these groups add their voices, it will help us. In our schools, it should be added to our curriculum, “…” it should be broadcast to everybody…*

Focus Group 4 (women 35+) participant: *I think it is very necessary for there to be more advocacy, it is also necessary for doctors and others to educate us.*

Schools, churches, community leaders, CSOs, and celebrities were all identified as actors with the potential to play a role in effective advocacy. In two of the groups (focus group 3 and 4), some participants said they planned to take the learning from the session back to community leaders/local churches to call for more advocacy.

Focus Group 3 (men 35+) participant: *We have a committee in this community, so I will like “…” to go to the chief and present the message to him so that they will arrange for the people to be educated on these issues. It is very important,*

Focus Group 4 (women 35+) participant: *I will take it home and tell my people. I also think the churches should take up “…” issues like this, the government should also help so that this information “…” gets to the village dwellers, because the ignorance is too much. NGOs [non-governmental organisations] should also get on board to spread the message.*

Participants’ desire to proactively share the information they encountered in the focus groups with their wider communities suggests there is substantial public appetite for campaigns to raise awareness of the health harms caused by unhealthy products, especially sugary drinks, which is where awareness seemed lowest. This desire for policy action to reduce the harms caused by these products was also evident in additional proposals that participants put forward, including restrictions on marketing or advertising and subsidies for healthier alternatives:

Focus Group 3 (men 35+) participant: *I can’t… understand how a country which have a mix of religious bodies that are against alcoholism… allows the advertising of alcohol on television, and these commercials are on peak times, when even children are watching TV…*

Focus Group 3 (men 35+) participant: *Some investors are willing to pay more taxes and still produce harmful products, so the government should “…” also make [gives examples like organic fruit juices] cheaper than the more harmful ones.*

Interestingly, the most frequently proposed alternative to health taxes—proposed in four of the five focus groups (all except focus group 1)—was to ban or limit harmful products (especially tobacco). Indeed, the proposals put forward by focus group participants were generally far more radical than the proposals that policy stakeholders in Ghana appear to be considering (Singh *et al* 2023).

### Popular frames within health tax debates

In analysing our data, we identified seven distinct frames relating to health taxes in Ghana within focus group discussions (far more than the two contrasting frames that we had initially exposed participants to). Table 3 summarizes each of these seven frames, with illustrative extracts and an assessment of the foundational basis of the frame (e.g. ‘fairness’), while the text that follows discusses how each frame appeared to impact on support for health taxes in Ghana and/or the specific design of such taxes.

**Table 3.** Public ways of framing health taxes.

Frame	Illustrative extracts	Foundational basis (e.g. moral basis)	Frequency in focus group data
The protective frame: Health taxes help protect young people and children from the harms arising from unhealthy commodity products.	Focus Group 4 (adult women) participant: ‘*in this country that we are living in there are thing that are going on that is very worrying, you will see a fine young boy who have been drinking so much that he has become useless, so if the taxes are placed on these products and it will prevent them from drinking I think it will help.’* Focus Group 5 (adolescent boys) participant: ‘*The youth play a key role in this because the youth, we are the future leaders, so if we say it is not good, which means it is not good, and will make sure our kids will not even take it, so we play a role’*	Societal responsibility to protect vulnerable populations (moral imperative)	Evident on multiple occasions in all 5 focus groups
The polluter pays frame: The industries that produce unhealthy commodity products behave in an unethical way, and this is a problem that should be addressed via health taxes.	Focus Group 1 (adolescent boys) participant: ‘*It is the same beverage companies, this alcohol people, who are sponsoring these keep fits and festivals […] They are deceiving us to think of the money side, but they are carrying our mind away from our health, and making us, make use of our money to buy their drinks, and to patronize their drinks, so I think they should also be checked, because our health is more important than the money that they are making,’* Focus Group 3 (adult men) participant: ‘*the problem here is the business tycoon who are making the huge profits out of it have their representatives in government, the people that they have bought their they conscience so that they can help them…’*	Fairness (ethical)	Evident on multiple occasions in all 5 focus groups
The ‘save money’ frame: Health taxes discourage people from purchasing unhealthy products, thereby enabling them to save money.	Focus Group 1: ‘*They should put the taxes on it, because the cigarette and the alcohol are all not good for our health so they should go ahead and tax them as a means to stop people from taking them. These will help us to also save our money.’* Focus Group 2: ‘*The taxes will help me stop buying and save money, as I am addicted to it if I don’t get the money to go and buy it will help me in so many ways.’* Focus Group 3: ‘*I want the taxes to be on these products because these will help prevent the abuse which will make us save money for other projects.’* Focus Group 5: ‘*I like sugar a lot, and all these products contains a lot of sugar, so for me, I think when the Taxes are high on it, I’ll stop or reduce the consumption rate, and that will help me save money, so it will transform my life in such a way, it will make me stop spending on things that I have no benefits from.’*	Financial gain and capability/development (personal gain)	Evident across several extracts in four focus groups (1, 2, 3 & 5)
The lifesaving imperative frame: Health taxes are a way of ‘Saving lives’ (achieving better health)	Focus Group 3 (adult men) participant: ‘*I support health taxes because it may help save lives.’* Focus Group 5 (adolescent boys) participant: ‘*For me, it will help me save money, and also save my life because if you take the alcohol, you will spend more money on hospital bills, but if they put more taxes on, it you stop taking the alcohol, and that will save your life. and save you from spending at the hospital, and give you a healthy life in the future’*	Saving lives (moral imperative)	Evident across several extracts in three focus groups (3, 4 & 5)
The ‘win-win’ for health frame: Health taxes are a ‘win-win’ policy that promotes health both through reduced consumption and via increased revenue for health care spending	Focus Group 3 (adult men) participant: ‘*I think raising the taxes is ok, because if they stop creating the problems, the burden on the government for health related problems will go down and the health of the people will get better, so it is a win, win situation.’* Focus Group 5 (adolescent boys) participant: ‘*I believe it’s good and it is very, very fair to do that, because the underlining word is health—health taxes will help in relieving certain things, and it will also help the people to reduce their consumption, which will help the government to reduce the spending on health, and it will help the government to save more funds, so that it can use to build more facilities for us’*	Improving lives (moral imperative)	Evident across several extracts in three focus groups (3, 4 & 5)
The nationalist frame: Health taxes help protect Ghana from harmful, externally produced products that are being imported.	Focus Group 3 (adult men) participant: ‘*I want to base my argument on these local drinks, we should try to change to that one and stop these foreign products,’*	Protecting Ghanaians (nationalist)	Briefly evident in Focus Group 1 but mentioned several times in Focus Group 3
The protect innocent bystanders frame: Health taxes help ensure people who do not consume unhealthy products are not as negatively impacted by these products.	Focus Group 4 (adult women) participant: ‘*you may not smoke cigarette but others may smoke and you who is standing by will be inhaling the smoke, but if it is not there at all there will be no secondary smoking, that is why I am saying the government should stop the companies from producing them to stop the smoking, or the taxes should be raised very high to make the prices unbearable.’*	Fairness (ethical)	Only evident in Focus Group 4

The way in which health taxes were framed appears to have implications for both group support and, in some cases, the kinds of health tax design that might have most popular appeal. For example, while the first two frames (‘protective’ and ‘polluter pays’) seem to imply general support for health taxes, both the ‘saving lives’ and the ‘win-win’ frames were premised on two assumptions: (i) that health taxes would both reduce consumption and increase revenue; and (ii) that this revenue would focus on health spending. Meanwhile, the ‘nationalist’ frame was predicated on an assumption that local products are healthier than imported products (this was largely applied to SSBs), with the implication that import taxes should be a key focus for any new health taxes. While the ‘bystander’ frame suggests that harms caused to those other than the consumer seem particularly unfair, which implies health taxes should focus on smoked tobacco products and alcohol, since participants suggested that it is these products that have more obvious impacts on bystanders.

We can also identify distinctive foundations for these different frames. Three of the frames (‘protective’, ‘lifesaving’, ‘win-win for health’) imply that a public health basis is persuasive, with some of the accounts suggesting that there is a moral imperative to implement taxes which can save lives. Two other frames (‘polluter pays’ and ‘protect innocent bystanders’) are grounded in a broader sense of fairness. The two remaining frames each have an alternative basis. The ‘save money’ frame is premised upon the importance of personal financial gain (usually to enable more meaningful spending), and the ‘nationalist’ frame has a distinctive focus on furthering the economic and health interests of Ghana as a country. Looking across the ways health tax frames were used and discussed by focus group participants provides a sense that certain types of tax designs would be more likely to appeal to members of the public than others. For example, taxes designed to reduce consumption of unhealthy products, to restrict the influence of unethical companies, to increase revenue for health spending, to promote local products, and to reduce negative externalities, seem most likely to engender public support. Reflecting this, the four outcomes that participants seemed most keen for health taxes to achieve are summarized in Table 4.

**Table 4.** Participant preferences for target outcomes of health taxes.

Desired outcome	Illustrative extract	Frequency in focus group data
Raise money for healthcare spending (especially for rural areas)	Focus Group 1 (adolescent boys) participant: ‘*I support the idea that government want to use the money for building health facilities I still support that all this product should be eliminated because our health is more better and expensive than what the government is going to get from the taxes…’* Focus Group 2 (adolescent girls) participant: ‘*I will support because my elder sister gave birth to a premature child at the Lapaz community hospital, but hey had no incubator, so they have to take the child to the regional hospital whilst she was on admission at the Lapaz hospital so she has to commute between the two hospitals, so if the government is going to use the money for the health facilities I will go for it.’*	Proposed in all five focus groups
Encourage manufacturers to make healthier produced (product reformulation)	Focus Group 2 (adolescent girls) participant: ‘*I think they should stop adding sugar in the drinks and also stop or reduce the amount of alcohol in the drinks,’* Focus Group 3 (adult men) participant: ‘*we have our local drinks like Sobolo* [a local drink made from Hibiscus flower petals]*, Lamogin* [another local, non-alcohol drink, made with ginger and rice] *and so on, the taxes should be placed on these products and should be very high, in other to force the manufactures to change into producing other products than these sugar things.’*	Proposed in all five focus groups
Restrict/tax advertising/marketing	Focus Group 3 (adult men) participant: ‘*place more taxes on commercials on these bad products’*	Focus Groups 3 and 4
Restrict harmful products coming into Ghana	Focus Group 3 (adult men) participant: ‘*I think we should just make it punitive in bring in these sugars into the country at the harbor, by taxing it very heavily,’*	Only in Focus Group 3

Of the potential health tax targets set out in Table 4, the most consistently popular proposal was for health taxes to be designed to raise revenue for health care spending. This was linked to widespread support for the idea that new health taxes should be earmarked for health spending. For example:

Focus Group 3 (men 35+) participant: *I know the effect of these products on the health of the people so “…” I will say they government should put heavy taxes on all three and earmark the revenue for improving health.*

While most participants appeared to support the idea of earmarking for health spending or, at least, did not raise objections, there was one exception. In Focus Group 3 (men 35+), one participant cautioned that easily available health care services might lead people to take less care of their health, though he also reasoned that if such measures were accompanied by education campaigns, this could be mitigated.

Focus Group 5 participants also discussed whether new (or additional) health taxes should be high and sudden or incremental and lower—and there appeared to be mixed views about this. When tax rates were discussed in the context of their potential to reduce consumption, very high taxes were proposed. However, when discussed in the context of the potential impacts of taxes on local businesses and businesses offering employment, it was suggested that taxes should not be too high. The following extracts capture this divergence:

Focus Group 5 (men 16-34) participants: *I believe it should be very, very, very high because when companies shut down, it literally saved a life and that is very good.*

“…”

*I think if there’s too much increase in the health taxes, it will shut down some companies and it will reduce the taxes that the government will get, so I think it shouldn’t be too much high.*

This suggests that framing not only impacts public support for health taxes, in principle, but also influences people's perceptions of the suitability of different tax rates.

## Discussion

Introducing new health taxes, or increasing existing ones, is a potentially attractive tool for governments facing fiscal and health spending pressures, as is currently the case in Ghana. The appeal of health taxes is reflected in Ghana’s decision to introduce a tax on SSBs in 2023, which diversified the range of national taxes contributing to NCD prevention, building on earlier decisions to tax tobacco (2004) and alcohol (2017). Ghana is now one of only a small number of African states levying taxes on SSBs (Laar *et al*. 2023). Yet, health taxes are also a form of government intervention that impact on people’s ability to make particular choices and can mean some products become unaffordable to those on lower incomes. As such, the expansion of health taxes is necessarily a deeply political matter in which debates about evidence are interwoven with ethics and values (Koon and Marten 2023). Given this, it is essential to ensure that health taxes have democratic support. Our study makes three key contributions to understanding public support for health taxes in Ghana.

Frist, our interviews with policy stakeholders in Ghana suggest there is a widespread belief (within government, industry and CSOs) that public support for health taxes, whether for new health taxes or for increases in existing taxes on unhealthy products, is limited, especially on more widely consumed products. This perception is important for health tax advocates to understand because it may inform hesitancy in maintaining existing health taxes and/or considering further health taxes. Yet, both recent opinion poll survey data (Laar *et al*. 2023) and our focus group discussions with members of the public in Accra suggest there is high public support for health taxes in Ghana. Furthermore, while policy stakeholders tended to believe that people were less likely to support taxes that they would be personally impacted by, focus group participants tended to express greater support for taxes that they believed would achieve greater health gains, notably more widely consumed products such as SSBs. This raises questions as to why policy stakeholders, selected on the basis of their expertise regarding health taxes, hold beliefs about public views that diverge from evidence of public views. It suggests that it may be useful for researchers to consider both how malleable public opinion is in response to work by competing interests (e.g. industry and health advocates) and to probe the empirical basis of claims made about public opinion within relevant media and policy debates. Further research could also usefully explore how the power asymmetries between ‘public’ and ‘private’ interests shape public views (Jennifer and Robert 2021), including by analysing media coverage of health taxes.

The second contribution of this study relates to unpacking how health taxes are framed in public discussion and the resulting consequences. As Koon and Marten’s (2023) scoping review demonstrates, framing not only shapes problem definitions and solutions but also engages moral judgments and social values, influencing public perception and political action. The semantic shift from ‘sin’ taxes to ‘health’ taxes is an example of efforts to change that way these taxes are interpreted (Koon and Marten 2023). Existing analysis identifies three common health tax frames: a health frame (taxes help promote health); a positive economic-fiscal frame (taxes help generate revenue for public spending); and a negative economic-fiscal frame (taxes negatively impact on national and personal finances) (Elliott *et al*. 2022, Koon and Marten 2023). While the first two frames are likely to reinforce public support, the third framing, which industry frequently promote (Koon and Marten 2023), may reduce public support. We know less about the malleability of public views in response to contrasting frames and evidence, or about the moral foundations of these frames (Koon and Marten 2023). The findings from our focus groups support existing evidence which suggest that taxes are more positively received when framed as ‘health’ interventions, with the ability to both reduce health problems and increase health spending (i.e. ‘win-wins’) (Wright *et al*. 2017, Elliott *et al*. 2022). This way of framing health taxes implies there is a moral imperative on governments to act to save lives and improve people’s health. Additionally, the focus groups identify five other frames that seem to increase public support for health taxes, with participants positively responding to the following frames, each of which was introduced by other participants: the need to protect children and young people; the importance of ensuring companies that profit from harmful products have to pay a price (similar to the ‘polluter pays’ frame often used in carbon tax debates—Dominioni 2022); taxes as an incentive for people to save money; taxes to protect Ghana from harmful, external influences; and the case for protecting people who do not consume health-harming products from these harms (e.g. passive smoke inhalation). The foundational basis for these additional frames varies from a moral imperative to protect vulnerable populations, to a sense of fairness, to personal financial gain (see Table 3), suggesting there is scope for those making a case for health taxes to employ a wider range of frames.

The third contribution of this paper, in the context of the dominance of opinion surveys, is to highlight the potential for achieving reasoned public discussion about health taxes via deliberative focus group discussions. This involves communicating available evidence to participants and encouraging public discussion. In addition to identifying new ways of framing health taxes, the focus group discussions demonstrated sophisticated engagement with the potential details of how new health taxes might work in a Ghanaian context, and insightful exchanges as to what was (and was not) considered ‘fair’ by different participants. Participants also signalled awareness of potential political barriers, such as political-business links, and made a case for advocacy. Indeed, a key strength of our paper is to demonstrate to researchers and funders the kind of in-depth insights into public views of health taxes that can be gained via this methodological approach.

A limitation of our deliberative focus group design is that, in seeking to enabled reasoned, evidence-informed discussion, we did not expose participants to the extent of industry-led framing and lobbying that is likely to inform media coverage of new health tax proposals in practice. This decision partly reflects the unique position that governments are in, when introducing new (or increased) health taxes, to provide an empirical rationale and to promote ‘win-win’, health frames. However, it was also an ethical decision in that we did not feel, as public health researchers, that it was ethically justifiable to strongly promote the perspectives of health harming industries to members of the public. Future research could usefully explore both how relevant industry actors in Ghana seek to shape public opinion around health taxes (and what role framing plays) and how they portray public opinion in media and policy debates (which would require comprehensive media analysis).

## Conclusions

Our findings suggest that, at least from the perspective of public opinion, there is potential to introduce new health taxes or increase existing ones in Ghana. Importantly, our findings identify a potential misalignment between the perceptions that policy stakeholders (people working in relevant parts of the government, industry and health CSOs) have of public opinion and the views that members of the public hold, which seem far more supportive of health taxes than stakeholders anticipate (at least when health taxes are explained). While this may mean that the rather pessimistic perceptions of policy stakeholders is misplaced, it may also represent an awareness among policy stakeholders that any health tax expansion is likely to meet well-resourced opposition from affected industries (Lauer *et al* 2023, Singh *et al* 2023). Since evidence consistently finds that affected industries work hard to prevent health taxes (Wright *et al*. 2017, Elliott *et al*. 2022), any proposal for expanding health taxes should be carefully designed to maximize health gains, and proactively framed in ways that increase popular support. Framing health taxes as interventions that will save lives and which represent a ‘win-win’ for health does seem to increase public support but, to be effective, may depend on a commitment to investing at least some of the revenue in health spending (Wright *et al*. 2017). Our research identifies further health tax frames that may increase public support (Table 3), including frames that promote the protection of particular groups (notably children and young people) and Ghana as a country, a frame centring on ensuring profiting industries ‘pay’ for harms caused, and a frame promoting health taxes as a means to incentivize personal financial savings. As such, the innovative deliberative insights presented in this paper help expand the framing ‘toolbox’ available for campaigns to promote health taxes.

## Supplementary Material

czaf042_Supplementary_Data

## Data Availability

The data underlying this article cannot be shared publicly due to the terms of the consent agreement, which offered participants anonymity as the full transcripts include details that could identify some individuals. Further aspects of the data will be shared on reasonable request to the Principle Investigator and co-author, Arti Singh (artisingh_uk@yahoo.com).

## Reflexivity statement

We are an international authorship team, made up of a mixture of genders, ethnicities and nationalities, across a range of career stages. While the lead author of this paper is based in the UK, reflecting the fact she led on drafting this paper, two co-authors are based in Ghana, where the research was undertaken, including the project lead, who is the last author here, reflecting her role as principal investigator overseeing the project (the last author was also the lead author of another paper from the same project).
