# Supplementary material for: New health taxes in Ghana: a qualitative study exploring potential public support
Source: Health Policy Plan. 2025 Jun 30;40(8):831–42. doi: 10.1093/heapol/czaf042 (PMC12448797; doi:10.1093/heapol/czaf042)
Supplement: czaf042_Supplementary_Data [file czaf042_supplementary_data.docx]

**Supplementary File**

**Overall objectives of the larger study**

This study aims to provide new evidence on the political economy challenges and opportunities associated with new health tax proposals in Ghana, and to explore how these can be overcome and harnessed to improve population health:

Our specific objectives are to:

1. To assess (via interviews and document analysis) the current state of readiness/interest among key government actors – particularly the Ministry of Health and Ministry of Finance in Ghana – for the use of consumption taxes on unhealthy products as a means of pursuing health policy goals.

2. To understand policymakers’ perceptions of the health, economic and political benefits and risks of such taxes.

3. To examine, through deliberative focus groups, public perceptions of existing taxes on unhealthy commodities, the potential for such taxes to play a role in improving, or addressing threats to, population health, and how such taxes would be perceived if they were re-designed so as to better advance health policy goals.

4. To explore, through the focus groups, how the public is likely to respond to earmarking of taxes for a range of activities, including: (i) enhanced public health services – e.g. tobacco and/or alcohol cessation services; (ii) increased allocations to the National Health Insurance Scheme, and (iii) increased allocations to primary care services.

5. To examine, again through the focus groups, the extent to which the public would regard earmarking commitments as credible; and how perceptions of credibility might be enhanced, e.g. through new legislative mechanisms, monitoring by independent bodies, scrutiny by supreme audit institutions etc)?

6. To examine commercial sector views of health taxes – including earmarked taxes - with the aim of better understanding the positions and preferences of major companies within key sectors (alcohol, tobacco and sugar-sweetened beverages).

7. To examine CSO/NGO perceptions and views on the potential of various options for health taxes in Ghana, with a view to ascertaining the extent to which a supportive ‘advocacy coalition’ for new health taxes might emerge in policy debates.

**Key Informant Interview Guide (Policy Stakeholders)**

Good morning/afternoon Mr/Mrs (Surname). Thank you for taking the time to meet with me today, I really appreciate it. I would like to start by formally introducing myself and briefly explaining the purpose of my research project and what you can expect from today’s interview.

My name is _____________________. Our team is conducting a research in collaboration with the WHO; to gain a better understanding of the perceptions of various stakeholders in health taxes on tobacco, alcohol and sugar sweetened beverages in Ghana in order to provide new evidence to review the current taxes and identify challenges and opportunities associated with new health tax proposals to improve overall health of the population Ghana. It is important to understand stakeholder’s perception and recommendations. This information is necessary for implementing future policies of the commercial determinants of health and for assisting the Ghana government to combat over nutrition and under nutrition during the current economic climate.

The reason I am interviewing you today is because you are a subject matter expert within your sector, and I believe that I can learn a lot from both your knowledge and experience.

Today’s interview will not take more than 45-60 minutes of your time. I would prefer if today’s interview could be very open, conversational and informed. As you are the professional, I would like to learn as much as possible from you. The interview is, and will remain, completely confidential and anonymous – your name and company will not be identified in the research report. As such I will not be referring to you as by your real name during the interview.

I would like to now ask for your permission to audio-record our conversation; I will be using both a recorder/phone as recording devices. Before we begin, I would like to ask if you could please read through and sign the informed consent form, including the section on consenting to being audio-recorded.

Do you have any questions with regards to today’s interview before I proceed?

[Terminology: 1 We define health tax proposals as proposals to introduce or increases existing taxes on health-damaging products in pursuit of explicit population health objectives.

For the purpose of today’s interview, I will be shortening sugar-sweetened beverages to SSBs.]

1. Demographics (hard copy for the participant)

1.1 Gender – Male / Female (Circle and say gender in audio-recording)

1.2 What is your date of birth?

1.3 What is your highest level of education?

1.4 What is your general job title?

1.5 How many years of experience do you have in your relevant field / sector?

1.6 Could you please talk me through what your role is in your sector?

1. There is a lot of discussion about health taxes but people sometimes mean different things – what do you think makes a particular tax a health tax?

• Can you briefly tell me about what you know on the current taxes on tobacco, alcohol and SSBs?

• How would you describe the current position on taxing alcohol, tobacco and SSBs in Ghana?

• Do you think it’s more important that a health tax achieves improvements in population health or provides revenue for government spending?

• How much government interest would you say there currently is in developing/increasing health taxes in Ghana.

Probe: Does this interest vary by Government Department?

2. Who would you say are the key stakeholders for health taxes in Ghana and how would you summarize their views?’

• How supportive of new/higher health taxes do you feel members of the public currently are?

Probe: How open to change do you think public views are for this issue?

• How supportive do you think key media outlets are of health taxes?

• Who are the key industry actors with an interest in health taxes in Ghana?

Probe: How influential do you think these actors are on the issue of health taxes?

• Who are the key CSO/NGO actors with an interest in health taxes in Ghana?

Probe: How influential do you think these actors are on the issue of health taxes?

• What about the interest of global actors (e.g. World Bank, WHO but also major donors) towards health taxes in Ghana?

Probe: How influential are these actors?

3. To what extent would you support the introduction and/or expansion of health taxes in pursuit of population health objectives? Prompts:

• What would you say are the advantages and disadvantages of health taxes from a government revenue perspective?

Probe: In general?

Probe: Specifically, in the COVID/post-COVID context in which public expenditure is set to fall sharply?

• What are the benefits of health taxes in achieving key health related outcomes?

• Are there any other countries that have experimented with health taxes that you are aware of?

Probe: Do you think there may be useful lessons for Ghana to draw on from those countries?

• To what extent do you feel there is a strong evidence-base for developing/expanding health taxes in Ghana?

o Are there any limitations of the current evidences that have?

4. What are the key barriers or challenges for introducing new/additional health taxes in the context of national political and societal dynamics?

• Political

• Administrative (e.g. would the MoF/MoH support this? Why?)Why not?

• Social

• Cultural

• Economic

• To what extent do you feel it is possible to overcome these challenges and what would need to happen to enable this?

5. What would you say the biggest opportunities for new health taxes in the context of national political, administrative and societal dynamics currently are in Ghana?

• Think of this in terms of earmarking them for National Health Insurance and/or government health services in the pursuit of health system goals, such as Universal Health Care

• Reduced demand for health-damaging goods

• Sustainable financing for public health services (e.g. in context of COVID?)

• Improvements to the physical environment

• Socio-economic status of population

6. How feasible would you say it is that Ghana develop and implement SSB taxes? (Ask CSO???)

• Administrative capacities and barriers

Probe: Does state capacity exist to implement such taxes effectively; if so, where; If not, how could it be built?

• To what extent might the legal framework present a challenge?

• Would it be feasible to generate sufficient public support?

• To what extent do you feel it would be feasible to manage industry interference/opposition?

7. What are your recommendations for the design of a new health tax in the context of Ghana?

• Would it focus on SSB or something else?

• Which kind of tax regime do you feel would be most likely to generate political, public, administrative, industry support? (ad valorem/specific/mixed or any other)

8. Do you have any alternative measures to reduce the rising non-communicable disease burden (obesity/diabetes/cardiovascular disease etc.) in Ghana?

Probe: Or any other ways to increase public funds that may help to support other health policy objectives?

**Schedule for Deliberative Focus Groups**

**Introduction to the project**:

- Thanks for participating
- Check consent form *(need an administrator/assistant on standby to help anyone who has issues with consent form)*

**Presentation 1:**

- Brief introduction to the project (aims, funding, timeline, expected outputs)
- The task for today’s discussion is to address the following: *“Evidence is clear that smoking, consuming alcohol and too much fat and sugar all have a negative impact on people’s health. Some people think that this means the government should try to limit people’s consumption of these products by increasing their price, via taxes, and using the money to fund better health services. Others think that taxing these products is unfair because it makes these products too expensive for some people and can worsen poverty. Should Ghana introduce new/additional health taxes on unhealthy products or not,* *and why?"*
- Briefly outline evidence demonstrating the extent of the problem (consumption of these products in Ghana and linked NCDs, life years lost, etc)
- Briefly outline evidence on need for additional healthcare spending in Ghana.
- Explain what we will do with the findings (e.g. present to policymakers and civil society actors in Ghana as well as the World Health Organization)

**Participant Introductions**

- Ask group to introduce themselves to the person next to them and explain that participants will then be introducing each other to the group. We are keen to find out people’s names and their immediate response to the task (supportive of health taxes of not?) [5 mins]

**Developing ‘conversation guidelines’**

Premise: “Everyone here has something to contribute; we want you to think about how best to help that happen – *What ‘conversation guidelines’ can help us to ensure that everyone can contribute and that everyone is heard?’* Support participants to draw up AND AGREE their own conversation guidelines for deliberation, with possible suggestions or challenges from facilitators. Facilitator/assistant records on flipchart.

**First set of questions**

1. Based on your own experiences and what you heard in the first presentation, if new health taxes were to be introduced in Ghana, do you think it should focus on tobacco, alcohol, sugary drinks or unhealthy processed food or something else?

- Facilitator to encourage different perspectives to come forward and to probe on why people hold the views they do.

1. How effective do you think taxes on unhealthy products are in changing people’s behaviours (e.g. alcohol taxes in reducing alcohol consumption, etc) and why? Do you think this differs depending on the product (e.g. cigarettes, wine, beer, sugary drinks, etc)?

- Again, facilitator should encourage different perspectives to come forward – if the group all agree, facilitator should give an alternative perspective and see how the group responds.
- Facilitator should probe on whether views vary by product.
- Where people don’t feel health taxes are effective, facilitator could ask what people feel would be more effective in reducing consumption.

**Facilitator intervention**: Facilitator to introduce debates around whether these taxes are fair or not (arguments for fairness: maintain choice, polluter pays principle; arguments unfair: make products unaffordable for poorer people and/or increase poverty).

**Second set of questions**

1. Has anyone changed their initial view about health taxes in any way? Were you persuaded by the evidence you heard that health taxes are effective in changing people’s behaviours – if so, why / if not, why not?

- Facilitator should probe on whether views vary by product (e.g. alcohol/tobacco/SSBs/unhealthy food) and also explore whether/why different kinds of evidence were more/less persuasive.

1. You have heard some contrasting views as to whether health taxes are fair. What do you think – are health taxes fair? If so, why? If not, why?

- Facilitator to encourage different perspectives and to re-introduce key aspects of the debate if participants aren’t bringing these in.

1. Do you think your support for a new health tax (or an increase on an existing health tax) would change if the government said all the money raised would be used to increase or improve healthcare in Ghana? If so, why? If not, why not?

- Facilitator to encourage different perspectives and to probe on whether it would make a difference extent to which trust in government commitments is an issue.

1. Has anything you’ve heard today changed how you think about health taxes?
2. Do you think civil society organisations focusing on health should be calling for more/new health taxes in Ghana? If so, why? If not, why not?
3. Do you think there should be rules on how external organisations (e.g. civil society organisations trying to promote health and industries that profit from unhealthy products) are able to influence policy discussions about health taxes in Ghana? If so, Why?
4. So far, we have used the term ‘health taxes’ but other people prefer the term ‘sin taxes’. What do you think about these two different terms? Does anyone prefer one term more than the other and, if so, why?

If the group all agree, facilitator should give an alternative perspective and see how the group responds.

**Thanks and follow-ups:** Facilitator to thank everyone and explain where people can find out more information about the project findings.

**Slides from presentation provided at the start of focus group sessions:**
